# Supplementary material for: Did the severity of appendicitis increase during the COVID-19 pandemic?
Source: PLoS One. 2022 Feb 10;17(2):e0263814. doi: 10.1371/journal.pone.0263814 (PMC8830628; doi:10.1371/journal.pone.0263814)
Supplement: Attachment — Submitted filename: Revised cover letter.doc [file pone.0263814.s001.doc]

**Author’s point-by-point *response* to review comments** (PONE-D-21-07736R1) :

We have carefully revised the manuscript in view of the editorial and reviewers’ comments as outlined in detail below.

**Reviewer’s comments**:

**Reviewer #1**: The authors have completely addressed the comments, and added response to text and study limitation.

*1. However, reference 4 and reference 10 are repeated. Need revision.* ***Response:*** Thanks to reviewer’s comment. We have updated references (by deleting repeated reference 10 and updating numbering of references) in the resubmitted manuscript.

**Reviewer #2:**  The quality of the paper has improved significantly with revision. The authors have addressed many of the concerns from the initial review, however I have some additional concerns on the revised manuscript.

Major comment

*1. As mentioned in the previous review, there are serious issues with this method from a statistical perspective due to the large number of regression models required. Although the authors do mention these limitations in the Discussion, there should also be a clear statement in the Methods that no adjustment was made for multiple comparisons. I would also recommend an explicit mention of the small sample size relative to the number of parameters included in the model, leading to imprecise estimates for some variables. Additionally, I believe the paper would benefit greatly by providing a comparison of the overall difference in moderate or severe appendicitis over 6 months between the study groups. This is a simple, less underpowered analysis which is important to provide context to the readers about the “overall” effect of the pandemic and to frame the results provided in the bi-week approach.*
***Response:*** Yes*,* we thank the reviewer’s informative comment.

A. We have added a statement regarding *no adjustment was made for multiple comparisons* in the Methods resection of the revised manuscript. *“*No adjustment was made for multiple comparisons when the sample size relative to the number of parameters was small.*” (last 3rd sentence, last paragraph, Materials and methods section)*

B. We have added a statement regarding *mention of the small sample size relative to the number of parameters* in the Methods resection of the revised manuscript. *“*No adjustment was made for multiple comparisons when the sample size relative to the number of parameters was small.*” (last 3rd sentence, last paragraph, Materials and methods section)*

C. We have also added *comparison of the overall difference in moderate or severe appendicitis over 6 months between the study groups* in the Results section of the revised manuscript. *“*Although the percentage of uncomplicated or complicated appendicitis was lower higher in the study group during the COVID-19 pandemic than that of the control group (68.5% vs. 58.9%), the difference was insignificant (P = 0.213). For comparison, the percentage of uncomplicated or complicated appendicitis over 6 months’ period between the study and control groups was 66.9 % (103/163)*.” (2nd and third sentences, 2nd paragraph, Results section).*

Minor comments

*1. Abstract: “The mean age was 46.2 + 19.8 years; male predominance was observed was observed (52.1%, 160/307).”
‘Was observed’ is repeated twice. ‘Predominance’ is not the right word in this sentence as there are only slightly more males than females.*
***Response:*** Yes*,* we appreciate the reviewer’s comment. We have delete “*; male predominance was observed was observed (52.1%, 160/307)*” in the revised manuscript.

*2. Materials and Methods, Dependent Variable: “The dependent variable was uncomplicated and complicated appendicitis.”
Suggest moving this sentence to after explanation of the different levels of appendicitis, and refining so it’s clear that this is a binary variable e.g. “The dependent variable was a binary indicator of uncomplicated and complicated appendicitis (0 – normal, mild; 1 – uncomplicated, complicated).”*
***Response:*** Yes*,* we thank the reviewer’s comment. We have deleted the 1st sentence *(dependent variable paragraph, Materials and methods section)* and added a sentence in the revised manuscript. *“*The dependent variable was a binary indicator of uncomplicated and complicated appendicitis (0 – normal, mild; 1 – uncomplicated, complicated).*” (5th sentence, dependent variable paragraph, Materials and methods section)*

*3. Materials and Methods, Statistical Analysis: “Patient characteristics of (excluding age), treatment (excluding surgery time), and disease variables aree reported as numbers and percentages.”
There are typos in this sentence – ‘of’ should be removed before “(excluding age)” and ‘aree’ should be replaced with ‘are’.*
***Response:*** Yes*,* we thank the reviewer’s comment. We have corrected these typos in the revised manuscript.

*4. Results: “Compared with the control group, the number of surgeries for appendicitis was relatively low between January 29 and April 21 in the study group (Figure 2).”
I would disagree with this statement, in my reading I see the numbers as approximately equivalent for the 25th of March to the 21st of April. Therefore, to avoid this subjectivity in the results section, I suggest reporting the actual number of surgeries in this period for either group rather than just referring to the figure.*
***Response:*** Yes*,* we thank the reviewer’s comment. We have added the actual number of surgeries in this period for either group in the revised manuscript. *“*Compared with the control group (70/158, 44.3%), the number of surgeries for appendicitis was relatively low between January 29 and April 21 (49/149, 32.9%) in the study group (Figure 2).*” (6th sentence, 1st paragraph, Results section)*

*5. Results: “Surgery was performed under general anesthesia in approximately 80% of the patients.”
Should replace ‘approximate’ with ‘approximately’.*
***Response:*** We appreciate the reviewer’s comment. We have made a correction in the revised manuscript.

*6. Results and Table 1: “The mean operation time was 67 + 27 min (range: 20–167 min). Dirty or contaminated wounds were observed in a quarter (25.1%, 77/307)”
The upper limit of the operation time is 168 minutes in Table 1 – please fix this so the values match. Table 1 also has a value of 7/307 for dirty or contaminated wounds, which looks like a typo.*
***Response:*** Yes*,* we appreciate the reviewer’s comment. We have made corrections for these typos in the revised manuscript.

*7. Results: “Compared with the control group, the percentage difference of uncomplicated and complicated appendicitis in the study group (vs. the control group) increased by more than 40% during the fifth bi-week (February 26–March 10) and eighth bi-week.”
Yes, but raw numbers of presentations must also be reported in this sentence to provide context to the percentage change. In both bi-week 5 and 8, the percentage change seems to be primarily driven by having fewer mild/normal cases rather than an increase in uncomplicated/complicated appendicitis. Providing the raw numbers in addition to percentage difference is necessary for transparency in the text as it was for Figure 3.*
***Response:*** Yes*,* we thank the reviewer’s comment. We have provided the raw numbers in addition to percentage difference in the revised manuscript. *“*Compared with the control group, the percentage difference of uncomplicated and complicated appendicitis in the study group (88.9%, 8/9 vs. the control group 42.9%, 6/14) increased by more than 40% during the fifth bi-week (February 26–March 10) (88.9%, 8/9 vs. the control group 42.9%, 6/14) and eighth bi-week (April 8–April 21) (90.0%, 9/10 vs. the control group 46.7%, 7/15) (Figure 3)*” (last sentence, 2nd paragraph, Results section)*

*8. Results and Table 2: “April 8–April 21 (OR = 11.25, 95% CI: 1.17–>100 , P = 0.036)”
Please report the upper limit of the 95% CI as a value, not ‘>100’. This also applies to bi-week 5 in the study group multivariate analysis.*
***Response:*** Yes*,* we thank the reviewer’s comment. We have reported these values in the Results and Table 2 of the revised manuscript.

*9. Results: “However, a such increase in OR for the severity of appendicitis was not observed in the univariate or multivariate analyses of the control group.”
This is not accurate for bi-week 2 as the estimated OR in the control group is 2.81 (univariate) and 6.38 (multivariate) which is quite similar to the ORs estimated in the study group (3.25 (univariate) and 5.81 (multivariate)). I believe the authors are discussing the 95% CI in this case, but the comment implies that the magnitude of the effect is small for the control group and so is inappropriate for this specific comparison.*
***Response:*** Yes*,* we appreciate the reviewer’s comment. From Table 2, we never found the ORs *(for the uncomplicated/complicated appendicitis)* over 7 in the right two columns of the control group *(neither univariate nor multivariate)*, which P values all insignificant. However, we noted that many ORs *(for the uncomplicated/complicated appendicitis)* overpass 7 in the left two columns of the study group *(univariate & multivariate)* and many of them were significant. Still, we have rephrased this sentence to minimize misunderstanding in the revised manuscript. *“*However, such a significant increase in OR (bi-weekly) for the severity of appendicitis was not observed in the univariate or multivariate analyses of the control group.*” (last 2nd sentence, last paragraph, Results section)*

*10. Discussion and Conclusion:
I believe the authors need to further review/soften some of the language around the findings from their study considering the limitations of the statistical methods used. For example:
- “This occurred when the number of confirmed cases of COVID-19 surged.”
- “The severity of appendicitis also increased, which inversely paralleled the number of surgeries for appendicitis during the pandemic.”
- “Therefore, during the COVID-19 pandemic, the number of surgeries for mild appendicitis has decreased, and the severity of appendicitis has increased.”
- “The number of patients with acute appendicitis seeking surgical treatment decreased as confirmed cases of COVID-19 surged.”
These kind of conclusions overstate the findings as a number of the ORs during the pandemic period in the study group have 95% CIs which are less than 1 (e.g. bi-week 6, 7 and 9). I recommend these sentences are amended to indicate the uncertainty evidenced in the 95% CIs.*

***Response:*** Yes*,* we thank the reviewer’s comment. We have modified these sentences in the revised manuscript.

*“***This probably occurred when the number of confirmed cases of COVID-19 surged in Taiwan.** In addition, **the severity of appendicitis also increased, which appeared to inversely paralleled the number of surgeries for appendicitis during the pandemic.***” (4th and 5th sentences, 1st paragraph, Discussion section)*

*“***Therefore, during the COVID-19 pandemic, the number of surgeries for mild appendicitis has seemed to decreased, and the severity of appendicitis has increased.***” (last sentence, 1st paragraph, Discussion section)*

*“***The number of patients with acute appendicitis seeking surgical treatment might decreased as confirmed cases of COVID-19 surged,…….***”(2nd sentence, Conclusions section)*
